# The Influence of Mesotrione on Human Colorectal Adenocarcinoma Cells and Possibility of Its Toxicity Mitigation by Cichoric Acid

**DOI:** 10.3390/ijms25115655

**Published:** 2024-05-22

**Authors:** Agata Jabłońska-Trypuć, Urszula Wydro, Elżbieta Wołejko, Monika Kalinowska, Grzegorz Świderski, Rafał Krętowski, Monika Naumowicz, Paweł Kondzior, Marzanna Cechowska-Pasko, Włodzimierz Lewandowski

**Affiliations:** 1 Department of Chemistry, Biology and Biotechnology, Faculty of Civil Engineering and Environmental Sciences, Bialystok University of Technology, Wiejska 45E Street, 15-351 Białystok, Poland; u.wydro@pb.edu.pl (U.W.); e.wolejko@pb.edu.pl (E.W.); m.kalinowska@pb.edu.pl (M.K.); g.swiderski@pb.edu.pl (G.Ś.); p.kondzior@pb.edu.pl (P.K.); w-lewando@wp.pl (W.L.); 2 Department of Pharmaceutical Biochemistry, Medical University of Bialystok, Mickiewicza 2A, 15-222 Białystok, Polandmapasko@gmail.com (M.C.-P.); 3 Department of Physical Chemistry, Faculty of Chemistry, University of Bialystok, Ciolkowskiego 1K, 15-245 Białystok, Poland; monikan@uwb.edu.pl

**Keywords:** mesotrione, cichoric acid, Caco-2 cells, oxidative stress, apoptosis, cancer

## Abstract

Mesotrione, as a widely used herbicide, is present in the environment in detectable amounts, causing serious damage. Here, we aimed to investigate the effect of mesotrione on Caco-2 cells and the possibility of its toxicity mitigation by cichoric acid. Therefore, we analyzed the cytotoxicity of both these compounds and the selected oxidative stress parameters, apoptosis and interaction of both the tested compounds with the cell membrane and their accumulation within the cells. In cytotoxicity studies, the stimulating activity of mesotrione was observed, and simultaneously, the inhibitory effect of cichoric acid was noticed. This effect was related to the results of oxidative stress analysis and apoptosis measurements. The activity level of key enzymes (glutathione peroxidase, catalase and superoxide dismutase) in Caco-2 cells exposed to cichoric acid was higher as compared to that of the control. The treatment with mesotrione did not induce apoptosis in the Caco-2 cells. The penetration of the studied compounds into the Caco-2 cells was measured by using an HPLC methodology, and the results indicate mesotrione’s high penetration capacity. The distribution of charge on the surface of the cell membranes changed under the influence of both compounds. Considering the mutual interactions of beneficial and potentially toxic food ingredients, it should be noted that, despite the observed favorable trend, cichoric acid is not able to overcome the toxic and cancer-stimulating effects of this pesticide.

## 1. Introduction

The intensive increase in agricultural production and, as a consequence, huge amounts of herbicides used, have resulted in the deterioration of soil quality, a lower quality of agricultural products, and increased emissions of pollutants, threatening the safety of the food chain [1]. An example of a widely used herbicide is mesotrione (Mes). Due to its high activity, low toxicity and physicochemical properties, including the ease of dissolving and mixing, it is widely applied in agriculture. It is mainly used for annual broadleaf weeds and the pre-emergence and post-emergence of grasses in corn fields. Moreover, mesotrione, due to its mobility in soil, can migrate to the surface water and ground water. According to the literature data, the half-life of mesotrione is 84 days in an aqueous solution, 3.4–4.8 days in plants (maize), and in soil, it could range from 6 to 21 days [2,3,4,5,6]. According to the PPDB, mesotrione is characterized by a moderate environmental effect, but at the same time, it is a compound with highly toxic effects on human health [7]. 

According to its chemical structure, mesotrione is [2-(4-methylsulfonyl-2-nitrobenzoyl)-1,3-cyclohexanedione]. This compound is isolated from the plant species *Callistemon citrinus*, and it was developed and originally marketed by Zeneca. It inhibits the enzyme 4-hydroxyphenylpyruvate dioxygenase. This enzyme is part of the biochemical pathway, which converts the amino acid tyrosine into plastoquinone and α-tocopherol, which are metabolized by plants [8]. In addition to its beneficial weed control properties, mesotrione also acts on non-target organisms with an additional toxic and damaging effect. Due to its poor adsorption in soil, mesotrione tends to leak into ground water during maize cultivation, with negative consequences for aquatic ecosystems [9]. There are few studies assessing the biodegradation of mesotrione and the relationship of this herbicide with ROS (reactive oxygen species) formation and damage in bacterial cells. Mesotrione transformed by *Bacillus* sp. 3B6 generates metabolites, 4-methylsulfonyl-2-nitrobenzoic acid (MNBA) and 2-amino-4-methylsulfonylbenzoic acid (AMBA), which have been found to be more toxic as compared to the parent compound [10]. According to Du et al., mesotrione and its metabolites cause algal blooms, and as a consequence, the widespread use of mesotrione may become an ecological problem because of the presence of its residues in soil and in water [11]. 

In our previous research, we focused mainly on the in vitro activity of Mes on various human breast cancer cells, melanoma cells and healthy human fibroblasts. We demonstrated the cytotoxic and carcinogenic effects of Mes [12,13,14]. However, the main route by which herbicides enter the human body is through the gastrointestinal tract. Therefore, we decided to analyze the effect of mesotrione on Caco-2 cells derived from the digestive system. The Caco-2 cells were originally obtained from a human colon adenoma (large intestine), and they are characterized by adherent growth. Caco-2 cells show many morphological and biochemical similarities to intestinal cells—enterocytes. The main feature that distinguishes Caco-2 cells from other cell lines is the ability to create a brush border, i.e., a system of microvilli on the cell surface. In addition, these cells have tight connections with each other (similarly to enterocytes), and they also have the ability to produce enzymes (e.g., alkaline phosphatase, sucrase and aminopeptidase) and systems, which transport substances from the lumen of the gastrointestinal tract directly into the bloodstream. As a result, they show a functional similarity to the epithelium of the small intestine, imitating the natural in vivo conditions of the gastrointestinal tract. The specific properties of Caco-2 cells result from the ability of malignant cells to differentiate into cells that are morphologically and physiologically similar to normal, intestinal cells (enterocytes) under the influence of inducing factors [15,16]. Due to these specific properties, the Caco-2 cell line is an excellent in vitro model for studying the effects of xenobiotics on the human body. 

Due to the toxic effects of pesticides on non-target organisms, it seems important to study the interactions between pesticides and food ingredients, such as polyphenolic compounds. Polyphenols may be a factor supporting the elimination of the toxic effects of pesticides. It is a fairly well-studied group of compounds in this respect, but many poorly known compounds from this group are still being analyzed. One of them is cichoric acid. Cichoric acid (CA) is obtained from plants of the *Asteraceae* family, e.g., *Cichorium intybus*, *Bidens tripartita* L., *Echinacea purpurea* and *Cichorium endivia* L. *C. intybus*; these are perennial herbs that are a rich source of cichoric acid and have been cultivated as high-quality vegetables since the 19th century. They can be cooked into salads and used for health-related purposes [17]. *Lactuca sativa* is an annual vegetable plant widely grown in temperate areas around the world. The content of chicory acid in different varieties varies significantly depending on different storage conditions, but this vegetable is also a rich source of cichoric acid [18]. *Ocimum basilicum* L. is a perennial herb cultivated all over the world as an aromatic cultivated and ornamental plant. Other studies have shown that the content of chicory acid in dried samples ranges from 0.09 to 0.16 mg/g [19]. In the above-mentioned plants and many other food products of plant origin, cichoric acid is a biologically active ingredient. Some researchers created in vitro and in vivo models that indicate that this compound improves people’s health due to its anti-inflammatory effects, maintenance of glucose and lipid homeostasis, neuroprotective properties, antioxidant properties and stimulation of the immune system. Its antiviral properties are also known, especially against the immunodeficiency virus, herpes simplex viruses and respiratory syncytial virus [20,21,22]. The ability to lower uric acid levels by *Cichorium intybus* is related to the presence of CA [23]. In the HepG2 cell line, CA has been shown to stimulate glucose uptake via glucosamine and to inhibit inflammation through NF-κB signaling [24]. In the synovial tissues of the ankle joint, CA can significantly reduce the levels of nuclear factor-κB (NF-κB), TNF-α and cyclooxygenase-2 (COX-2) [25]. In our previous studies, we have also demonstrated the double effect of CA; it is beneficial in relation to normal cells and cytotoxic in relation to cancer cells [12,26,27,28]. 

Taking into account the results of our previous research indicating the undeniable cytotoxicity and carcinogenic properties of all the pesticides we have tested, it seems crucial to find a food ingredient that is able to eliminate or minimize the toxic and carcinogenic effects of pesticides, the presence of which in food seems inevitable. Therefore, the aim of this study was to assess the carcinogenic potential of mesotrione and the cytotoxic potential of chicory acid and to analyze the mechanisms of action of both the tested compounds in intestinal adenocarcinoma cells. We wanted to investigate the potential effectiveness of CA as an agent to reverse the toxic effects of mesotrione. To achieve the above goals, it was crucial for us to analyze cytotoxicity, and then analyze the molecular mechanisms of action of mesotrione and CA in relation to their activity in the apoptosis and oxidative stress pathways. Therefore, considering the interplay between the beneficial and toxic food components, we wanted to investigate and present their possible interactions in the most suitable Caco-2 cell model.

## 2. Results

### 2.1. Cytotoxicity

**Figure 1:**
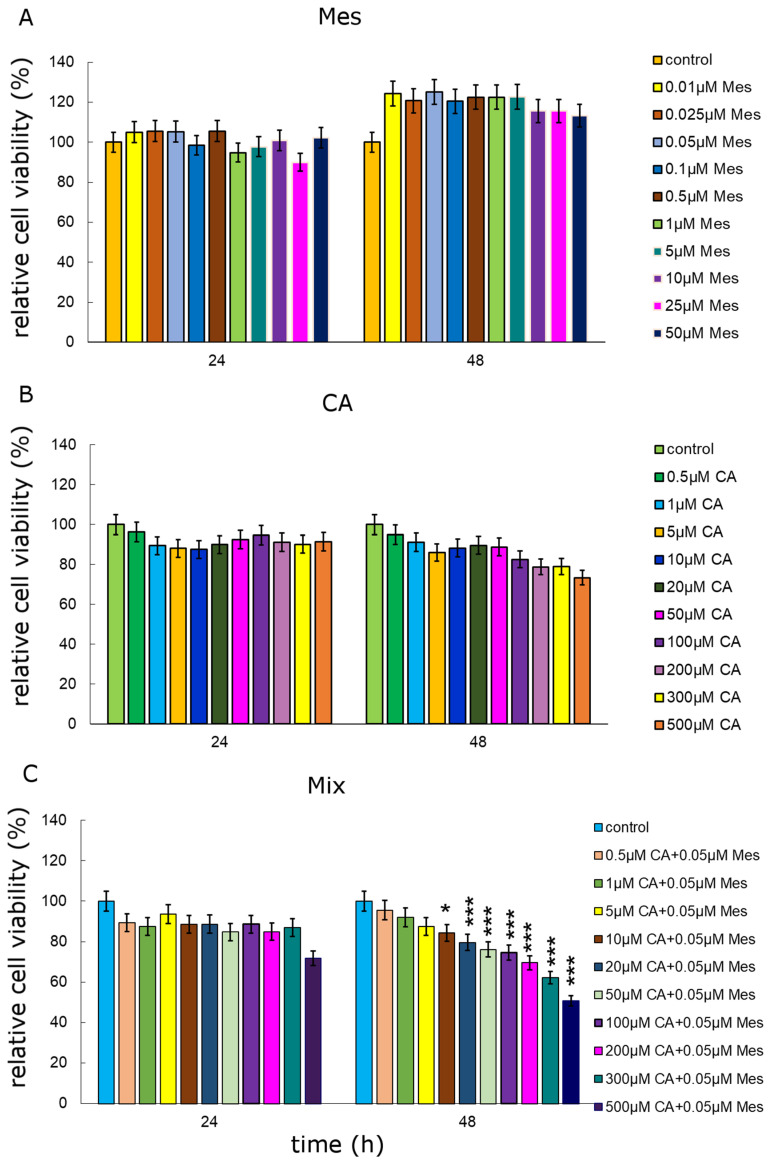
Cell viability results for Caco-2 cell line exposed to different concentrations of CA (cichoric acid) (**A**), Mes (mesotrione) (**B**) and a mix of the two compounds (**C**) for 24 h and 48 h calculated as a percentage of control untreated cells. Each value on the graph is the mean of three independent experiments, and error bars show the standard deviation (SD). * *p* < 0.05, and *** *p* < 0.001 represent significant effects between the treatments and control followed by a Dunnett’s test.

In order to estimate the relative cell viability and the potential cytotoxicity of the studied compounds, an MTT assay was applied (Figure 1). CA caused a decrease in the relative viability of Caco-2 cells, which was observed just after 24 h of treatment. At a concentration of 10 µM CA, a 12% decrease was observed after 24 h of treatment. However, 48 h of incubation caused the highest decreases in the viability of neoplastic cells. Concentrations of 300 µM and 500 µM reduced cell viability by about 20% and more than 26%, respectively, after 48 h of treatment. CA at any concentration tested did not stimulate cell viability. In contrast, Mes significantly increased the relative cell viability after 48 h. The most intense growth stimulation, of about 25% as compared to that of the control, was observed under the influence of 0.05 µM Mes, as is shown in Figure 1B. Considering the results obtained in the MTT test, a Mes concentration of 0.05 µM was selected for further analysis. This concentration of Mes was used together with the entire range of CA concentrations. A significant decrease in the relative viability of the cells was noted, even by 50%, especially in the combination of Mes with CA at 0.05 µM + 300 µM and 0.05 µM + 500 µM, respectively (Figure 1C). After analyzing the obtained results, one combination of both the compounds was selected for further analysis of the mechanisms of their action in Caco-2 cells. The highest concentration of CA, 500 µM, was close to the IC50 value. In order to investigate the parameters of oxidative stress and apoptosis, the effect of 0.05 µM Mes + 500 µM CA was analyzed.

### 2.2. Oxidative Stress

**Figure 2:**
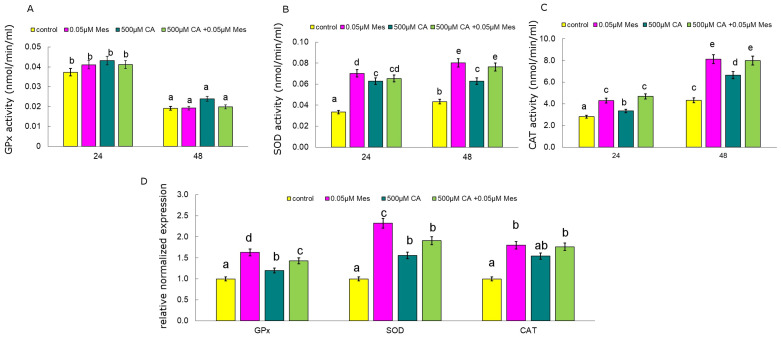
The influence of CA, Mes and the mix of CA and Mes on catalase activity (**A**) GPx (glutathione peroxidase) activity (**B**) and SOD (superoxide dismutase) activity (**C**) and on GPx (glutathione peroxidase), SOD (superoxide dismutase) and CAT (catalase) relative gene expression (**D**) in Caco-2 cells. The cells were cultured with 500 µM of CA, 0.05 µM of Mes, and a mix of 500 µM CA + 0.05 µM Mes for 24 h and 48 h. Mean values from three independent experiments ± SD are shown. Different letters indicate statistical differences (*p* ≤ 0.05) between each treatment estimated by Tukey’s test.

The influence of Mes and CA and the mixture of Mes with CA on the expression of selected genes encoding antioxidant enzymes, GPx (glutathione peroxidase), SOD (superoxide dismutase) and CAT (catalase), in the Caco-2 cell line was studied (Figure 2). It was observed that application of Mes and the mix of 500 µM CA and 0.05 µM Mes significantly increased the expression of the *GPx genes* as compared to that of the control. In turn, the results obtained for the *SOD gene* showed that in each studied variant, the expression of this gene was significantly higher than that in the control, but the highest value was obtained for the cells exposed to Mes. Similarly, the expression of the *CAT gene*, both in the samples treated with Mes and CA in the variants as single substances as well as their mixture, was significantly higher than that in the control.

The first line of antioxidant defense, which plays a key role in maintaining redox homeostasis in the cell, are enzymes, such as GPx, CAT, and SOD. A significant increase in catalase activity under the influence of CA in combination with 0.05 µM Mes was observed already after 24 h of incubation (Figure 2). CA at a concentration of 500 µM, used as a pretreatment before the addition of Mes, increased the catalase activity by approximately 18%, respectively, as compared to the untreated control cells. Mes at a concentration of 0.05 µM increased the catalase activity in both the treatment times and after 48h by about 87% as compared to that of the control. The opposite results were observed for glutathione peroxidase, as an increase in enzyme activity was noted after 24 h of incubation, and this is true for both the control and the cells treated with the test compounds (Figure 2). GPx activity was the highest under the influence of 500 µM CA in both the tested incubation times. Our results show that SOD activity was enhanced not only by the action of Mes, but also by CA. A significant increase in SOD activity as compared to that of the control was observed at both the tested times (Figure 2). 

**Figure 3:**
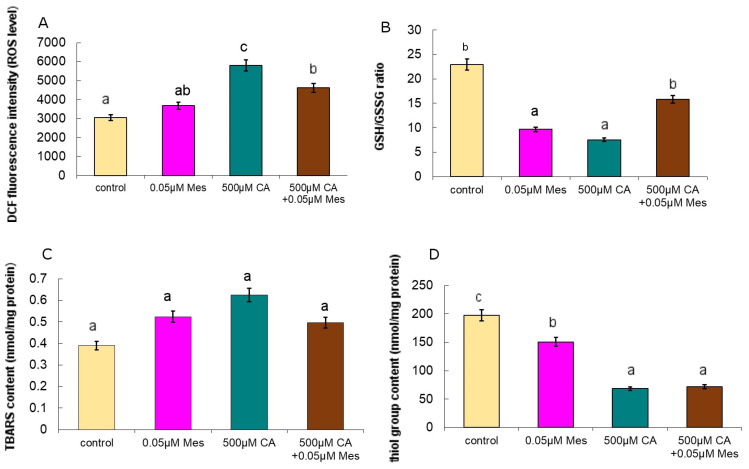
The effect of Mes, CA and a mix of CA with Mes on the level of intracellular ROS (**A**), the GSH/GSSG ratio (**B**), the TBARS content (**C**) and the SH group content (**D**) in the Caco-2 cells. The cells were incubated with 0.05 µM Mes, 500 µM CA and a mix of 500 µM CA + 0.05 µM Mes for 24 h. Mean values from three independent experiments ± SEM are shown. The different letters indicate statistical differences (*p* ≤ 0.05) between each treatment estimated by Tukey’s test.

Figure 3A shows the effect of CA and Mes on ROS production in the Caco-2 cells. The relative amount of ROS is shown as the fluorescence intensity of 2′7′-dichlorodihydrofluorescein (DCF) in the Caco-2 cells cultured with CA and Mes (Figure 3A). At a concentration of 500 µM, CA caused a significant increase of about 90%. The obtained results indicate the enhancing effect of CA on the formation of ROS, which is related to the lipid peroxidation process and apoptosis.

The effect of the tested compounds on the GSH/GSSG (reduced glutathione/oxidized glutathione) ratio is shown in Figure 3B. Reduced glutathione was found in a group of very important antioxidants that maintain oxidative balance in the cell. At the concentration of 500 µM CA, it significantly decreased the GSH/GSSG ratio, while 500 µM of CA in combination with 0.05 µM Mes significantly increased the tested parameter as compared to that of the CA-treated cells. When comparing the results to the control, a decrease in the examined parameter was observed in each case. Based on the obtained results, we conclude that both CA and Mes have an inhibitory effect on the GSH/GSSG ratio in the Caco-2 cell line.

Neoplastic cells usually are characterized by an increased level of oxidative stress, which is a factor that stimulates their proliferation. Among the many processes that generate free radicals, lipid peroxidation is one of the most important as it is an excellent source of free radicals (Figure 3C). Incubation with Mes caused an increase in the TBARS (thiobarbituric acid reactive species) content (Figure 3C), which was analyzed as an indicator of lipid peroxidation. CA caused a significant (over 60%) increase in the TBARS level as compared to that of the control, but 500 µM CA mixed with Mes caused an increase of about 27% as compared to that of the control. This can be explained by the presence of other lipid peroxidation products, e.g. HNE. 

The influence of Mes and CA on the level of SH groups (thiol groups) is shown in Figure 3D. After exposure to Mes, a statistically significant decrease in the content of thiol groups by about 23% was observed. The exposure to CA also caused a decrease in the analyzed parameter by as much as 65% as compared to that of the control. It is noteworthy that a decrease was also observed with the pretreatment of CA. The obtained results indicate that 500 µM CA eliminated the Mes stimulatory effect on the Caco-2 cells, as a decrease in the thiol content caused by 0.05 µM Mes in the culture pretreated with 500 µM CA was observed. The obtained results may indicate that CA may be a potential compound that intensifies oxidative stress in neoplastic cells, even in the presence of a herbicide. 

### 2.3. Apoptosis 

**Figure 4:**
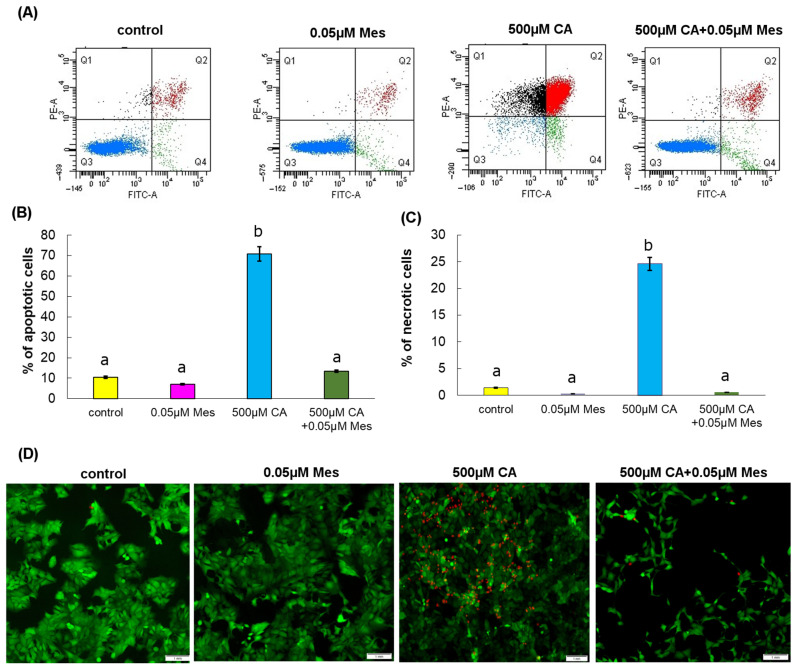
The effect of CA, Mes, and the combination of CA + Mes on apoptosis in Caco-2 cells, which were incubated with 500 µM of CA, 0.05 µM of Mes, and a mix of 500 µM CA + 0.05 µM Mes. (**A**) Bar graphs presenting the percentage of apoptotic cells (Q1 black – necrotic cells, Q2 red-late apoptotic cells, Q3 blue-live cells, Q4 green-early apoptotic cells). (**B**) Apoptotic cells and (**C**) necrotic cells. Representative dot plots for the 24 h treatment cells subjected to Annexin V-FITC/propidium iodide (PI) staining are shown. (**D**) The effect of Mes, CA and the mix of Mes and CA on apoptosis and necrosis in the Caco-2 cell line was evaluated by fluorescence microscope assay (200× magnification, scale bar 1 mm). The cells were incubated with 0.05 µM Mes, 500 µM CA, and a mix of the two compounds for 24 h and stained with calcein-AM and propidium iodide. We present representative images from one of three independent experiments. The mean values from three independent experiments ± SD are shown. The different letters indicate statistical differences (*p* ≤ 0.05) between the treatments estimated by Tukey’s test.

Apoptosis was assessed by using flow cytometry, and the results are shown in Figure 4. Figure 4 shows the percentage of apoptotic and necrotic cells incubated for 24 h with CA, Mes and with the mixture of CA and Mes. The values obtained with the herbicide treatment indicate that the test compound does not increase apoptosis. However, the treatment with CA, in particular alone, increased apoptosis significantly. 

In order to confirm the occurrence of apoptosis, a fluorescence microscopy assay was used (Figure 4). Calcein-AM only stained the viable cells, while propidium iodide stained the viable and dead cells. The morphology of apoptotic cells was assessed by fluorescent staining. As with flow cytometry analysis, we observed differences between the control treatments, CA and CA + Mes. Slight differences were observed between the control cells and the cells treated with the pesticide. Only CA caused the appearance of a much greater percentage of apoptotic cells. 

### 2.4. HPLC and Surface Charge Density of Cell Membranes

**Figure 5:**
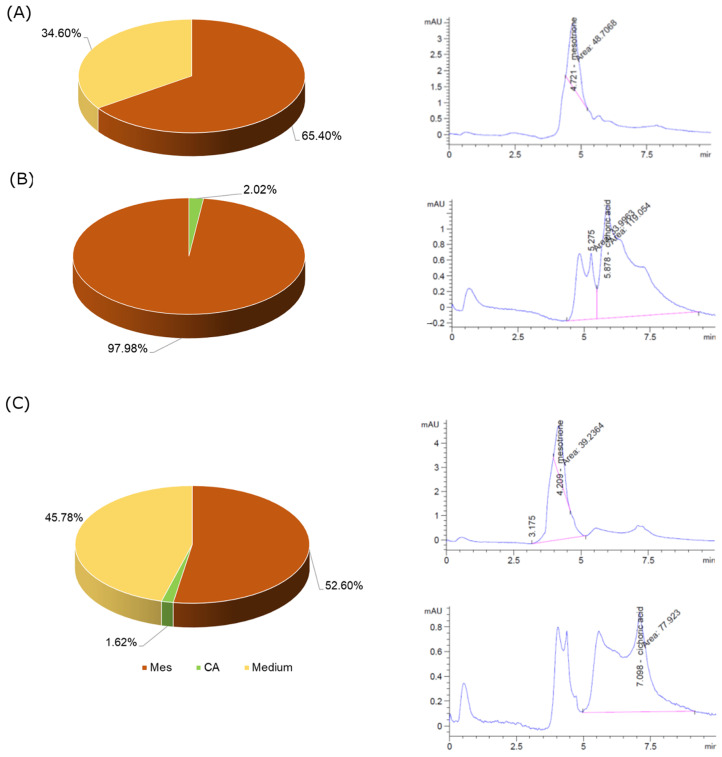
Percentage of Mes (**A**), CA (**B**) and CA + Mes (**C**) accumulation in Caco-2 cells estimated using HPLC methodology.

The results obtained from HPLC analysis confirm that Mes is a compound that is perfectly absorbed within the cell, and it penetrates the cell membranes of Caco-2 cells. On the other hand, CA, probably due to its chemical structure, penetrates the cells to a slight extent. The described effect was observed both in the case of exposure of cells to single compounds and to a mixture of both the tested compounds (Figure 5).

**Figure 6:**
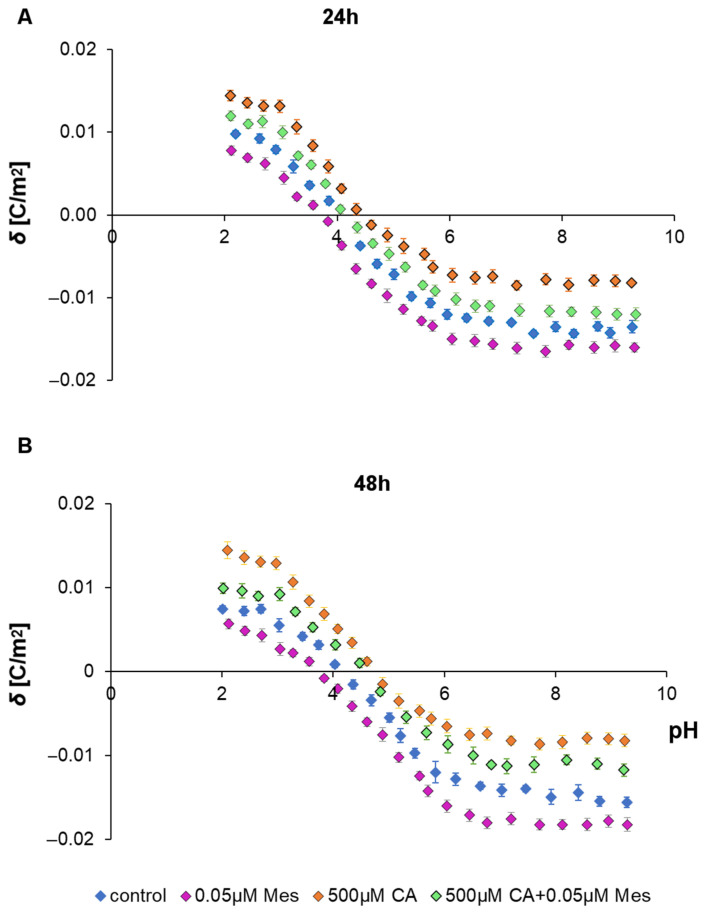
Dependence of surface charge density of Caco-2 cells as a function of the pH of the electrolyte solution. The cells were untreated (navy blue) or treated with Mes (grey), CA (orange) and Mes and CA in mixture (green) for 24 h (**A**) and 48 h (**B**).

In order to check if Mes, CA and the Mes + CA mixture interact with/adsorb on the cell membrane, microelectrophoretic mobility measurements were undertaken for the Caco-2 cells treated with these compounds for 24 h and 48 h. Analyses were carried out at pH values ranging from 2 to 9.5, with sodium chloride as a supporting electrolyte. The values of the surface charge density were calculated based on electrophoretic mobility according to Equation (1). For quantitative data, average values with standard deviations were reported. The pH-dependent changes in surface charge density of the Caco-2 cells are described as similarly shaped curves for all the studies (Figure 6). The treatment of Caco-2 cells with Mes caused an increase in negative charge at low/high pH values compared with that of the untreated cells. The isoelectric point of the Caco-2 cell membranes treated with Mes shifted to lower pH values compared with that of the untreated cancer cell membranes. Conversely, administering CA or the Mes + CA mixture to the Caco-2 cells caused a decrease in negative charge at low/high pH values compared with that of the untreated cell membranes. Moreover, it was found that the isoelectric point of the CA- or Mes + CA-mixture-treated Caco-2 cell membranes shifted to higher pH values compared with that of the untreated Caco-2 cell membranes.

## 3. Discussion

Due to the very widespread use of pesticides, both humans and animals, which are important links in the food chain, are constantly exposed to the side effects of their action [29]. Therefore, it is important to study the potentially protective effect of natural compounds derived from plants on the functioning of human cells. Interestingly, it was found that exposure to Mes may cause oxidative damage, which was confirmed in our previous experiments [12,13,14]. In this study, we attempted to reverse the cytotoxic effect of the fungicide by using a cytoprotective compound such as cichoric acid. 

The literature data indicate that phenolic compounds of plant origin are effective against tumor cell proliferation, but their bioabsorption is very poor. Therefore, some enhancers are being used such as chitosan [30]. However, CA activity against colorectal adenocarcinoma cells has not been extensively studied, especially in combination with Mes. In the first stage, the influence of the analyzed compounds on the proliferation of the Caco-2 cell line was analyzed using an MTT assay, on the basis of which the concentrations were selected for further analyzes. Statistically significant decreases in the viability of the Mes-treated cells were observed following the application of 500 µM of CA; however, it should be mentioned that after 24 h treatment of the Caco-2 cells with Mes and separately with CA, almost no statistically significant response was observed. Only the treatment with both the compounds caused a statistically significant effect right after 24 h of incubation. The results presented are in line with our previously published data on the effects of CA on various cell lines, including the DLD-1 line representing colorectal adenocarcinoma cells, in which we have shown a decrease in the proliferation and viability of CA-treated cancer cells [27]. Also, Huntimer E.D. et al. exhibited the influence of phenolics, among them cichoric acid, on the proliferation of cancer cells [31]. Generally, our results are consistent with the literature data, indicating the stimulation of cancer cells by various types of pesticide [32,33].

**Figure 7:**
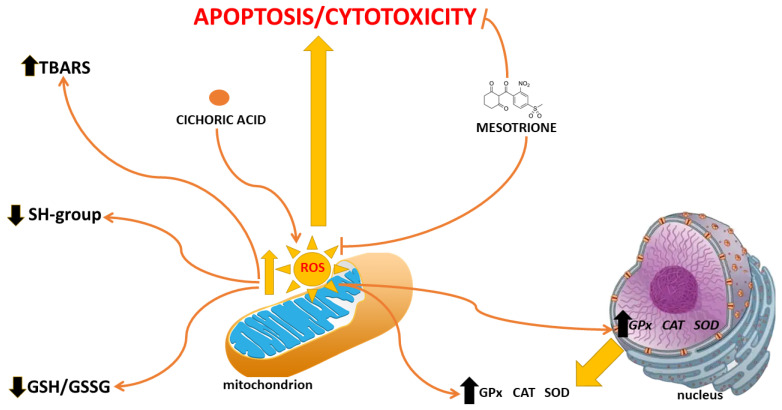
Tentative model of Mes and CA mechanisms of action in Caco-2 cell line.

There are a lot of literature data showing that pesticides stimulate the proliferation of neoplastic cells by acting through various mechanisms, e.g., bifenox and dichlobenil increase oxidative stress, while stimulating the proliferation of neoplastic cells and inhibiting apoptosis [34]. A relationship has been shown between an increased level of oxidative stress and the simultaneous increase in the proliferation of neoplastic cells [35]. Therefore, we focused primarily on the analysis of various parameters of oxidative stress, but also on the study of the process of apoptosis. As compared to normal cells, neoplastic cells are characterized by high levels of ROS and RNS, which are mainly due to their genetic disorders, which cause uncontrolled proliferation. CA exhibited significantly anti-proliferative properties in the Mes-treated cells. Neoplastic cell proliferation is inextricably linked with oxidative stress [36]. The obtained results indicate a significant increase in the level of ROS in the Caco-2 cells treated with CA, which may be related to the anti-proliferative activity of the studied polyphenolic compound (Figure 7). Neoplastic cells usually show a high level of oxidative stress and a high content of free radicals, the main function of which is to counteract the antioxidant defense [37]. The generation of a large amount of ROS in neoplastic tissues is a key stage in tumor progression [38]. Our research findings revealed the Mes stimulation of ROS production in Caco-2 cells, but the results include statistically insignificant and statistically significant increases in the level of ROS in the CA-treated cells at the same time. 

ROS are generated by the partial reduction of molecular oxygen to superoxides (O_2_^−^•), hydrogen peroxides (H_2_O_2_), lipid peroxides (ROOH), or the corresponding hydroxyls (HO•) and peroxyl radicals (ROO•). The toxic effect of lipid peroxides in cells is manifested by the existence of two main mechanisms of their action. Firstly, lipids are the key structural molecules that determine the integrity and proper functioning of cell membranes; therefore, their peroxidation results in disturbances in their structure and function [39]. Moreover, lipid peroxides are highly reactive molecules that promote further ROS generation and the damage of important structural macromolecules in cells, leading to a further increase in the TBARS content. One of the best studied effects of ROS on the structure and function of cell membranes in both healthy and neoplastic cells is lipid peroxidation. It is a natural metabolic process, by which lipid hydroperoxides, oxidized lipid peroxidation products and lipid peroxidation initiators such as ROS are formed. These molecules are involved in signal transduction, cell proliferation control and apoptosis [40]. Cancer cells are definitely more predisposed to macromolecule oxidation as compared to healthy cells. Our results revealed a similar dependency. In the Mes-treated Caco-2 cells, we noticed an increase in the TBARS content, which is correlated with a simultaneous decrease in SH groups (Figure 7). Changes in the level of TBARS and the SH group content were also accompanied by changes observed in the GSH/GSSG ratio. CA caused a decrease in GSH content, which can explain its anticancer activity. An elevated level of GSH has been found in many different types of tumor. This molecule is necessary for their growth and proliferation. Therefore, compounds which act by reducing its amount are considered to be antitumor. CA exhibits anticancer properties; however, when the cells under study were exposed to Mes, CA was unable to lower the GSH level sufficiently. An appropriate level of one of the most important antioxidants, GSH, usually correlates with cancer cell resistance to the induction of apoptosis and their increased proliferation [41]. Ibrahim KA et al. claimed that after exposure to the pesticide fenitrothion, the content of GSH significantly decreased [42]. Interestingly, our results were consistent with above-mentioned results, which suggested that this pesticide caused oxidative damage after exposition. The content of GSH in the cells is also reduced due to the excessive production of lipid peroxidation products and other reactive oxygen species. 

There are many indicators of homeostasis at the cellular level, among which changes in the level of oxidative stress are one of the most important [43]. The GSH/GSSG ratio, ROS content, MDA and thiol levels reflect the state of oxidative stress. When the body is exposed to external physical and chemical stressors, the ROS balance is disturbed, and then the activity of antioxidant enzymes is also disturbed [44,45,46]. Changes in the contents of GSH, MDA and selected antioxidative enzymes, such as catalase, glutathione peroxidase or superoxide dismutase activity within the cell, may cause an increase in the content of toxic by-products of lipid peroxidation, e.g., ROS and other free radicals. Their presence leads to oxidative damage, eventually causing various diseases [47]. In order to prevent oxidative damage, the breakdown of hydrogen peroxide into oxygen and water is catalyzed by CAT, which together with SOD form an antioxidant system [43]. Enzymatic antioxidants such as SOD, GPx and CAT belong to the endogenous, antioxidant defense system [36,48]. The presented results clearly show the induction of an increase in oxidative toxicity in the Caco-2 cells treated with both Mes and CA and a mixture of these compounds. This is evidenced by the observed increase in the activity of antioxidant enzymes and the increase in TBARS content. The excessive amount of ROS induced by Mes causes a significant decrease in GSH synthesis and stimulates the activity of antioxidant enzymes (Figure 7). After co-exposure with CA, the degree of damage was slightly alleviated, but not significantly in terms of the SOD CAT and GPx levels. The TBARS content can give rise to lipid peroxidation and intensifies membrane damage, which is the marker of oxidative stress. Glutathione peroxidase (GPx) is one of the isoenzymes, which use glutathione to reduce H_2_O_2_, lipid hydroperoxides and organics, thereby preventing oxidative damage. Our results revealed that the content of TBARS and the activity of GPx showed opposite trends after exposure to CA, which indicated that the reduction in TBARS content might be related to enhanced GPx activity. Increased GPx activity promoted TBARS removal. This conclusion was consistent with a study in which pesticide exposure reduced the content of MDA and increased the activity of GPx [49]. Cichoric acid increases the activity of antioxidant enzymes, and thus reduces the level of ROS, which is caused by the molecular mechanisms related to the enhanced nuclear translocation of nuclear factor erythroid 2-related factor 2 (Nrf-2) and the level of peroxisome proliferator-activated receptor-γ coactivator α (PGC-1α) [50]. Due to the fact that oxidative stress is closely related to the genesis and development of some types of cancer and many chronic diseases, the above results of our research suggest the potential future use of cichoric acid in the treatment of diseases associated with oxidative stress, in particular those generated by substances commonly found in food such as pesticides.

Oxidative damage is one of the crucial indexes of pesticide toxicity [51]. In order to further analyze the oxidative stress caused by Mes and CA in Caco-2 cells, we conducted molecular verification. In this study, the expression of SOD-, CAT- and GPX-encoding genes trended with the enzyme’s activity in the Caco-2 cells after co-exposure to CA and Mes. Therefore, it is possible that enzymes activity is greatly influenced by the environment. To support this idea, Fukai T. et al. reported that SOD is the first-line indicator of antioxidant status [52]. In the current study, the transcription of SOD-encoding genes changed significantly, and it speculated that SOD is the main regulatory enzyme of Mes-induced oxidative stress in Caco-2 cells (Figure 7). 

Changes in the level of oxidative stress are associated with the induction of apoptosis. In this study, apoptotic cells were detected by propidium iodide and calcein-AM staining. We used microscopic examination to confirm the results obtained from the cytometer. In comparison with the control group, the ratio of red fluorescence indicated extensive cell apoptosis in the CA-treated group, while Mes tended to delay apoptosis in the CA-exposed cells. Apoptosis could be induced either by the internal pathway or the external pathway [53]. The internal apoptotic pathway is mediated by proteins including the Bak, Bcl-2 and Bax families. These families can be provoked by plentiful signals, such as oxidative stress, growth factor detachment and cellular damage [54]. It should be emphasized that Mes and CA have a synergistic effect in inhibiting cell proliferation; however, this effect is not correlated with the induction of apoptosis. It should be noted that the inhibition of proliferation and the induction of apoptosis do not always have a direct correlation because the inhibition of cell proliferation may be due to the blockade of the cell cycle [55,56]. According to Tao H. et al., selected fungicides have the ability to block Caco-2 cells in the G2 phase of the cell cycle, and mitosis cannot occur, which subsequently affects proliferation [57]. However, we did not observe similar results, but completely different ones. Mesotrione did not stop, but intensified cell proliferation, without stimulating apoptosis. CA at a concentration of 500 µM significantly induced apoptosis, but not in combination with Mes. However, CA exhibits very weak cell penetration; therefore, its proapoptotic activity probably is the result of mechanisms other than those occurring inside the cell, probably related to the activation of the receptors on the cell membrane surface. This was confirmed by the results obtained from the analysis conducted using the HPLC method. CA penetrated the Caco-2 cells to a very low extent, in contrast to Mes, which penetrated the cell membranes to a statistically significant extent. Recently, it became apparent that some features of the cancer cell membrane should be highlighted in order to enhance drug development. These parameters, such as the membrane charge, the membrane lipid composition, membrane surface packing, membrane asymmetry and fluidity, appear to make a significant contribution to potential drug/membrane interactions. Hence, studies of these interactions are becoming significant tools to better understand the therapeutic activity of anticancer drugs and their toxic effects. Changes both in the surface charge density values and isoelectric point position induced by the treatment of Caco-2 membranes with Mes, CA and the mixture of both the compounds correspond to changes in the composition of cancer membrane functional groups, which may be due to the appearance or disappearance of new functional groups in a reaction with these compounds. These results warrant further investigation into the involvement of membranes in colorectal adenocarcinoma cell function. To sum up, the significant stimulation of cancer cells was observed as a result of their exposure to the tested fungicide, which is the result of the effective penetration of this compound through cell membranes and the stimulation of oxidative stress to a level supporting the proliferation of Caco-2 cells, while reducing the level of apoptosis. However, the second tested compound, cichoric acid, showed a much lower ability to penetrate the cell membranes and interact with them, which resulted in a weaker effect, especially if it was applied to the cells together with the tested fungicide (Figure 7). To sum up, it is worth noting that although the Caco-2 cell line is an excellent research model for the bioavailability of substances in vitro, it has certain limitations that may affect the quality of the obtained results. First of all, it is a cancer cell line, which has certain biological properties that distinguish it from the cell lines of healthy enterocytes. Caco-2 cells originate from the colon, not the small intestine, although phenotypically they resemble small intestinal enterocytes. It is worth mentioning that fully differentiated cells of the Caco-2 line are characterized by a high level of expression of genes encoding glucose (SGLT1 and GLUT2) and fructose (GLUT5) transporters, which are located in the jejunum in vivo. The hPEPT1 peptide transporter present in the ileum has also been identified [58]. To sum up, in vitro studies using cell cultures do not provide information on an individual’s response to a given chemical compound. Bioavailability is determined not only by the profile of enzymes and transporters, but also by the individual, characteristic features of the host; therefore, it is impossible to directly extrapolate in vitro data to the in vivo situation.

The obtained results of this study have a number of implications. They show that there are some interactions between beneficial and toxic food ingredients such as cichoric acid and mesotrione. It was also shown that a closer examination of cichoric acid’s possible effects as an anticancer agent is needed. Because of its poor ability to penetrate membranes, some enhancers of bioavailability are necessary. The results presented in this paper reinforce the idea that cichoric acid needs to be studied more closely for its interactions with pesticides and for its possible application in some clinical settings. 

## 4. Materials and Methods

### 4.1. Reagents

Dulbecco’s modified Eagle’s medium (DMEM) with 4.5 mg/mL (25 mM) of glucose with Glutamax, penicillin, streptomycin, trypsin–EDTA, FBS (Fetal Bovine Serum) and PBS (Phosphate-Buffered Saline) (without Ca and Mg) were provided by Gibco (San Diego, CA, USA). MTT reagent, cichoric acid, mesotrione and dichlorodihydrofluorescein diacetate (DCFH-DA) were purchased from Sigma-Aldrich Co. A GSH/GSSG-Glo™ Assay kit, SDS, TCA, TBA and Folin–Ciocalteu reagent were provided by Sigma-Aldrich, and DTNB was provided by Serva. A cell stain double-staining kit containing propionium iodide and calcein-AM was provided by Sigma-Aldrich, St. Louis, MO, USA. An RNeasy Mini Kit was provided by QIAGEN, Hilden, Germany. An iScript cDNA Synthesis Kit, PrimePCR ™ SYBR^®^ Green Assay and SsoAdvanced™ Universal SYBR^®^ Green Supermix were provided by Bio-Rad Laboratories GmbH, Munich, Germany. The GAPDH gene was provided by Genomed S.A. (Warsaw, Poland). An FITC Annexin V apoptosis detection Kit I was provided by BD PharmingenTM, San Diego, CA, USA.

### 4.2. Cell Culture

The influence of mesotrione (Mes) and cichoric acid (CA) was studied in the Caco-2 cell line, which was obtained from the American Type Culture Collection (ATCC, Manassas, VA, USA). The Caco-2 cells were cultured in DMEM (Gibco) supplemented with 10% FBS (Gibco), penicillin (100 U/mL) and streptomycin (100 μg/mL) at 37 °C in a humified atmosphere of 5% CO_2_ in air. 

The cell viability in the tested cell lines was examined at concentrations of 0.5 µM, 1 µM, 5 µM, 10 µM, 20 µM, 50 µM, 100 µM, 200 µM, 300 µM and 500 µM for CA and 0.01 µM, 0.025 µM, 0.05 µM, 0.1 µM, 0.5 µM, 1 µM, 5 µM, 10 µM, 25 µM and 50 µM for Mes. 

### 4.3. Chemical Treatment of Cells

CA and Mes were stored in a refrigerator at a temperature of 4 °C, and a stock solution was prepared by dissolving them in TrisHCl buffer. Compounds were added to the cultured cells for a final concentration in the ranges from 0.5 µM to 500 µM for CA and from 0.01 µM to 50 µM for Mes. The control cells were incubated without test compounds. 

### 4.4. Cichoric Acid and Mesotrione Cytotoxicity 

CA and Mes cytotoxicity was measured with the use of MTT reagent according to the method of Carmichael using 3-(4,5-dimethylthiazol-2-yl)-2,5-diphenyltetrazolium bromide (MTT) (Sigma-Aldrich, St. Louis, MO, USA) [12]. The Caco-2 cells were seeded in 96-well plate at a density of 1 × 10^4^ cells/well, and after 24 h time, to attach them to the plate surface cells, they were treated with CA in the concentration range from 0.5 µM to 500 µM, Mes in the concentration range from 0.01 µM to 50 µM, and finally CA mixed with Mes in the concentration range from 0.5 µM to 500 µM mixed with 0.05 µM Mes. After 24 h and 48 h, the cells were subjected to an MTT assay. Absorbance was measured with the plate reader, GloMax^®^-Multi Microplate Multimode Reader (Promega Corporation, Madison, WI, USA). Cell viability was presented as a percentage of control, untreated cells. This study was performed in triplicate to ensure consistent results were obtained.

### 4.5. RNA Extraction and cDNA Synthesis

Total RNA of the Caco-2 cell line was extracted by using the RNeasy Mini Kit (QIAGEN, Hilden, Germany) on the QIAcube System (QIAGEN, Hilden, Germany) according to the manufacturer’s protocol. The quality of RNA samples was controlled using the QIAxcel Advanced System (QIAGEN, Hilden, Germany). cDNA synthesis was performed from 1 µg of each total RNA sample using the iScript cDNA Synthesis Kit (Bio-Rad Laboratories GmbH, Munich, Germany) in accordance with the manufacturer’s instructions. The transcribed product was diluted at 1:10 and stored at −20 °C until further analysis.

### 4.6. Reverse Transcription-Quantitative PCR (RT-qPCR)

Gene primers encoding glutathione peroxidase (GPx), superoxide dismutases (SOD1) and catalase (CAT) in human cells were obtained from the Bio-Rad collection for the PrimePCR ™ SYBR^®^ Green Assay (Bio-Rad Laboratories GmbH, Munich, Germany). GAPDH was used as a housekeeping gene, and its sequence and properties were published by Piana et al. (2008) [58]. All the PCRs were performed in 20 µL reaction mixtures containing 1 µL cDNA (diluted 1:10), 10 µL SsoAdvanced™ Universal SYBR^®^ Green Supermix (Bio-Rad Laboratories GmbH, Munich, Germany), 1 µL PrimePCR Assay (for CAT, SOD1, GPx) or 0.5 µL of each reverse and forward primer of GAPDH (10 µM), and nuclease-free water to 20 µL. No template controls (NTCs) and negative RT samples (reverse-transcription-omitted) were used for every target gene. Each biological replicate was run in triplicate on a CFX96 Touch Real-Time PCR Detection System (Bio-Rad Laboratories GmbH, Munich, Germany) according to the following thermocycling protocol: polymerase activation step at 95 °C for 30 s, 40 subsequent cycles at 95 °C for 15 s, and 30 s at 60 °C, followed by melting curve analysis at 65–95 °C, with 0.5 °C increment at 5 s/step. The results were analyzed using CFX Manager Software Version 3.1 (Bio-Rad Laboratories GmbH, Munich, Germany). Transcript levels were calculated relative to the controls and are expressed as relative normalized expressions (2^−ΔΔCt^).

### 4.7. Catalase Activity

Catalase is involved in the detoxification processes, mainly in the metabolism of hydrogen peroxide. Catalase activity was studied according to Jabłońska-Trypuć et al. [12]. The cells were cultured in six-well plates at a density of 1 × 10^5^ cells/well (Sarstedt); subsequently, they were treated with 500 µM CA, 0.05 µM Mes, and a mix of two compounds (CA + Mes concentrations: 500 µM + 0.05 µM) for 24 h and 48 h. For the determination of catalase activity, the Catalase Assay Kit (Cayman Chemical Company, Ann Arbor, MI, USA) was used following the manufacturer’s instructions. The absorbance of final product was read at 540 nm using the GloMax^®^-Multi Microplate Multimode Reader. All the experiments were performed in triplicate.

### 4.8. Glutathione Peroxidase Activity 

Glutathione peroxidase plays an important role in cell protection against oxidative stress, catalyzing the reduction of hydroperoxides. Glutathione peroxidase activity was studied according to Jabłońska-Trypuć et al. [12]. The cells were cultured in six-well plates at a density of 1 × 10^5^ cells/well (Sarstedt), and they were treated with 500 µM CA, 0.05 µM Mes, and a mix of two compounds (CA + Mes concentrations: 500 µM + 0.05 µM) for 24 h and 48 h. For the determination of glutathione peroxidase activity, the GPx assay kit (Cayman Chemical Company, Ann Arbor, MI, USA) was used following manufacturer’s instructions. Absorbance was read at 340 nm using the GloMax^®^-Multi Microplate Multimode Reader. All the experiments were conducted in triplicate. 

### 4.9. Superoxide Dismutase Activity

Superoxide dismutases catalyze superoxide anion dismutation to molecular oxygen and hydrogen peroxide, and they are involved in the cellular antioxidant defense system. Superoxide dismutase activity was studied according to Jabłońska-Trypuć et al. [12]. The cells were seeded in six-well plates at 1 × 10^5^ cells/well (Sarstedt), and they were treated with 500 µM CA, 0.05 µM Mes, and a mix of two compounds (CA + Mes concentrations: 500 µM + 0.05 µM) for 24 h and 48 h. For the determination of SOD activity, the Superoxide Dismutase Assay Kit (Cayman Chemical Company, Ann Arbor, MI, USA) was applied following manufacturer’s instructions. Absorbance (440–460 nm) was read using the GloMax^®^-Multi Microplate Multimode Reader. All the experiments were conducted in triplicate.

### 4.10. Determination of GSH/GSSG

The total glutathione and GSH/GSSG ratios were each studied in triplicate using GSH/GSSG-Glo™ kit (Promega Corporation, Madison, WI, USA) following the manufacturer’s instructions. The cells were seeded in white bottom 96-well plates at 1 × 10^4^ cells/well (Sarstedt), allowed to attach, and treated with CA at a concentration of 500 µM, Mes at a concentration of 0.05 µM, and a combination of two compounds (CA + Mes concentrations: 500 µM + 0.05 µM). Prior to the assay, growth media were removed, and the cells were washed with PBS. This assay is based on a luminescence measurement and detects and quantifies the total glutathione (GSH + GSSG), GSSG and GSH/GSSG ratios in cultured cells. Stable luminescent signals are correlated with either the GSH or GSSG concentration of a sample. In this method, the GSH-dependent conversion of a GSH probe, Luciferin-NT, to luciferin by a glutathione S-transferase enzyme is coupled with a firefly luciferase reaction. Light from luciferase depends on the amount of luciferin formed, which, in turn, depends on the amount of GSH present. Thus, the luminescent signal is proportional to the amount of GSH. The GSH/GSSG ratios were calculated directly from luminescence measurements. Luminescence was read using the GloMax^®^-Multi Detection System. All the experiments were conducted in triplicate.

### 4.11. Total Protein Content in Cells

Adherent cells (2.5 × 10^5^ cells/mL) in 2 mL of culture medium were incubated with or without the test compounds in tissue culture 6-well plates. After the homogenization of Caco-2 cells and extraction in 0.1 M NaOH at 4 °C, the total protein content was calculated. The concentration of proteins was determined spectrophotometrically as per Lowry. Folin phenol reagent with a protein kit calibrated with bovine serum albumin as the standard was used in the experiment. The absorbance of extracts was measured spectrophotometrically at 750 nm [26]. All the experiments were conducted in triplicate.

### 4.12. Determination of TBA Reactive Species (TBARS) Levels

The level of TBA-reactive species (TBARS) as membrane lipid peroxidation markers was measured using the method of Rice-Evans et al. as described previously [26]. Adherent cells (2.5 × 10^5^ cells/mL) in 2 mL of culture medium were incubated with or without the test compounds in tissue culture 6-well plates. The cells were washed with PBS (pH 7.4), scraped from Petri dishes, and subsequently resuspended in 1 mL of PBS. TCA (15%, 1 mL) and TBA (0.37%, 1 mL) were added to 1 mL of the cell suspension and mixed. This mixture was submerged in a boiling water bath for 10 min and the concentration of TBARS was assessed spectrophotometrically at 532 nm using the extinction coefficient of 156 mM/cm. All the experiments were conducted in triplicate.

### 4.13. Determination of SH Groups

The SH groups were measured using the method of Rice-Evans (1991) as described previously [26]. Adherent cells (2.5 × 10^5^ cells/mL) in 2 mL of culture medium were incubated with or without the test compounds in tissue culture 6-well plates. The cells were washed twice with PBS (pH = 7.4; 4 °C) and dispersed by scraping. The Caco-2 cells were counted, resuspended in 1 mL of PBS, and collected by centrifugation at 5000× *g* for 10 min. The pellet was resuspended in 1 mL of 0.5 M phosphate buffer (pH 7.8) containing 0.1% SDS. Subsequently, 25 μL Ellman’s reagent (5 mM) was added, and the thiol groups were measured spectrophotometrically at 412 nm using the molar extinction coefficient of 13.6 mM^−1^ cm^−1^. All the experiments were conducted in triplicate.

### 4.14. Intracellular ROS Detection

The level of intracellular reactive oxygen species (ROS) was determined using dichlorodihydrofluorescein diacetate (DCFH-DA), (Sigma, St. Louis, MO, USA) [34]. After diffusion through the cell membrane, DCFH-DA is deacetylated by cellular esterases to a non-fluorescent compound, which is later oxidized by intracellular ROS into a fluorescent 2′, 7′–dichlorofluorescein (DCF). The Caco-2 cells (2 × 10^4/^well) were seeded in 200 µL of growth medium in black 96-well plates. After 24 h, the medium was removed, and the cells were stained with 10μM of DCFH-DA in PBS at 37 °C using a 5%CO_2_ incubator for 45 min. Next, the dye was removed and replaced with CA at a concentration of 500 µM, Mes at a concentration of 0.05 µM, and a combination of two compounds (CA + Mes concentrations: 500 µM + 0.05 µM) and incubated for 24 h. Then, the DCF fluorescence intensity was measured by using the GloMax^®^-Multi Detection System at the excitation wavelength of 485 nm and the emission wavelength of 535 nm. Intracellular ROS generation in the pesticide and the CA-stimulated Caco-2 cells is shown as the intensity of fluorescence of the DCF. All the experiments were conducted in triplicate.

### 4.15. Fluorescent Microscopy Assay

For apoptotic and necrotic cell nuclear morphology evaluation, fluorescent dyes such as propidium iodide and calcein-AM were used. The Caco-2 cells were seeded on cell imaging dishes with cover glass bottom with CA at a concentration of 500 µM, Mes at a concentration of 0.05 µM, and a combination of two compounds (CA + Mes concentrations: 500 µM + 0.05 µM) and without the tested compound as a control for 24 h. After incubation, the cells were washed twice with PBS, and then stained with the dye solution in the dark in 37 °C for 15 min. After incubation, the staining mixture was removed, and the cells were washed with PBS and analyzed by using a fluorescent microscope (200× magnification). Calcein-AM stained only the viable cells, and PI only passed through the disordered areas of the dead cell membrane. The cells were analyzed and photographed via using Olympus IX83 fluorescent microscope with SC180 camera with the Cell Sens Dimension 1.17 program. The following criteria were used, living cells with regularly distributed green chromatin nuclei were stained with a green color and dead cells that were probably apoptotic cells were characterized by red nuclei with chromatin condensation or fragmentation, while necrotic cells showed red-stained cell nuclei.

### 4.16. Detection of Apoptosis and Necrosis 

The apoptosis and necrosis of Caco-2 cell line were evaluated by flow cytometry on the FACSCanto II cytometer (BD, San Diego, CA, USA). The Caco-2 cells (2.0 × 10^5^ per well) were seeded in 2 mL of medium in six-well plates. After 24 h, the medium was removed, replaced with a CA concentration of 500 µM, Mes concentration of 0.05 µM, and a combination of two compounds (CA + Mes concentrations: 500 µM + 0.05 µM) in the medium. The cells were incubated for 24 h. The cells were detached, resuspended in the medium, and then in a binding buffer. Subsequently, the cells were stained with FITC Annexin V and PI (FITC Annexin V apoptosis detection Kit I, (BD PharmingenTM, San Diego, CA, USA) at room temperature in the dark for 15 min. The data were analyzed using FACSDiva software version 6.1.3 (BD PharmingenTM, San Diego, CA, USA).

### 4.17. HPLC-DAD Measurement

The obtained samples were filtered through SEPARA membrane filters (non-syringes) with PTFE membrane (pore diameter: 0.45 µm) and analyzed using the HPLC Agilent 1260 Infinity with a DAD detector. The chromatographer was equipped with a Zorbax Eclipse Plus C18 Analytical column (4.6 × 250 mm; 5 µm). The injected volume of samples was 20 μL. The mobile phase consisted of 50% acetonitrile (A) and 50% water (B) at a flow rate of 1 mL/min. The temperature of the thermostat was 40 °C. The identification was performed on the basis of retention time and the DAD absorbance spectra of the standards (λ = 260 nm for mesotrione; λ = 330 nm for cichoric acid). The content of chemical compounds was determined with a five-point calibration curve of standards in the following range of concentration: 0.01–0.05 µM (Mes) and 100–500 µM (CA).

### 4.18. Electrophoretic Light Scattering Measurements

The electrophoretic mobility of the Caco-2 cells was measured using a zeta potential analyzer (Zetasizer Nano ZS; Malvern Instruments Ltd., Malvern, UK) applying the electrophoretic light scattering technique. Disposable folded capillary cells (Malvern DTS 1070) were used to perform the experiment. All measurements were conducted as a function of pH with a WTW InoLab pH 720 laboratory meter (WTW, Weinheim, Germany).

The samples suspended in 0.9% NaCl solution were titrated to the required pH in the range from 2 to 9.5 with NaOH or HCl, and surface charge (*δ*) measurements were taken every ± 0.3 pH units. Six electrophoretic mobility measurements (each comprising 100–200 runs of 2 s duration) for every sample at a given pH value were carried out.

The surface charge density was determined by converting electrophoretic mobility values according to the following equation [59]:

(1) $$\delta =\frac{\eta \cdot u}{d}$$

where *η*—the viscosity of the solution; *u*—electrophoretic mobility; *d*—the diffuse layer thickness.

The diffuse layer thickness was determined using the following expression [60]:

(2) $$d=\sqrt{\frac{\varepsilon \cdot \varepsilon_{0}\cdot R\cdot T}{2\cdot F^{2}\cdot I}}$$

in which *R*—the gas constant; *T*—temperature; *F*—the Faraday constant; *I*—the ionic strength of 0.9% NaCl; and *ε* and *ε*_0_ refer to the permeability of the electric medium. 

### 4.19. Statistical Analysis 

All experiments were replicated at triplicate, and the data are presented as mean values ±SD (standard deviation). Differences between the treatments and untreated control human cells were analyzed by one-way ANOVA. When significant differences were detected between the means for cell viability after treatment with Mes, CA and the mix and the control, Dunnett’s test was used. Significant effects are represented by *p* ≤ 0.05 (*), *p* ≤ 0.01 (**) and *p* ≤ 0.001 (***). For the other determined parameters, Tukey’s HSD test was used for pairwise comparisons of means, with which homogeneous groups were formed. A significance level of *p* < 0.05 was applied. Statistica 13.3 package was used for analyses.

## 5. Conclusions

In this study, we showed that Mes even at a low concentration significantly increased the viability of Caco-2 cancer cells and increased oxidative stress, especially ROS production and the lipid peroxidation levels. Its effect turned out to be so strong that the cichoric acid used did not overcome the toxic effects of the pesticide. This indicates the effect of herbicides on the metabolism of cancer cells, their resistance to apoptosis, and consequently, the possibility of resistance to standard treatment regimens, which should be further investigated in the future. In particular, research on the impact of pesticides on the metabolism of cancer cells should focus on the molecular mechanisms of carcinogenesis and metastasis, which are the most dangerous from the point of view of human health. Moreover, understanding the key stages of carcinogenesis induction by pesticides will allow us to investigate which stages of the metabolic pathways should be inhibited to prevent cancer development. This, in turn, can be used in the analysis of biologically active compounds of plant origin, which could inhibit this process and effectively reduce the risk of cancer development. In future research, it is worth considering other active compounds from this group of plant polyphenols, which could prove more effective in combating the toxic effects of pesticides in the human body. At the same time, it seems crucial and justified to conduct such in vivo tests in the next stage, for example, using animals. This will certainly provide a broader picture and greater application possibilities of the obtained results.

## Data Availability

The data presented in this study are available on a reasonable request from the corresponding author.
